# Practical Blood Flow Restriction Training: New Methodological Directions for Practice and Research

**DOI:** 10.1186/s40798-022-00475-2

**Published:** 2022-06-28

**Authors:** Rodrigo Ramalho Aniceto, Leonardo da Silva Leandro

**Affiliations:** Study and Research Group in Biomechanics and Psychophysiology of Exercise, Department of Physical Education and Sport, Federal Institute of Education, Science and Technology of Rio Grande do Norte, Rua Manoel Lopes Filho, nº 773. Valfredo Galvão, Currais Novos, RN CEP: 59380-000 Brazil

**Keywords:** KAATSU training, BFR, Restriction pressure, Prescription, Elastic wraps

## Abstract

Most studies with blood flow restriction (BFR) training have been conducted using devices capable of regulating the restriction pressure, such as pneumatic cuffs. However, this may not be a viable option for the general population who exercise in gyms, squares and sports centers. Thinking about this logic, practical blood flow restriction (pBFR) training was created in 2009, suggesting the use of elastic knee wraps as an alternative to the traditional BFR, as it is low cost, affordable and practical. However, unlike traditional BFR training which seems to present a consensus regarding the prescription of BFR pressure based on arterial occlusion pressure (AOP), studies on pBFR training have used different techniques to apply the pressure/tension exerted by the elastic wrap. Therefore, this Current Opinion article aims to critically and chronologically examine the techniques used to prescribe the pressure exerted by the elastic wrap during pBFR training. In summary, several techniques were found to apply the elastic wrap during pBFR training, using the following as criteria: application by a single researcher; stretching of the elastic (absolute and relative overlap of the elastic); the perceived tightness scale; and relative overlap of the elastic based on the circumference of the limbs. Several studies have shown that limb circumference seems to be the greatest predictor of AOP. Therefore, we reinforce that applying the pressure exerted by the elastic for pBFR training based on the circumference of the limbs is an excellent, valid and safe technique.

## Key Points

- The literature demonstrates that in most cases the elastic wrap can replace the pneumatic cuff traditionally used for BFR training.
- It is suggested to use 10–30% elastic restraint percentages based on the limb circumference at rest for pBFR training, depending on the type of elastic, experience and training objective, and also thinking about the individual's safety and adherence to the training program.
- Future studies should be conducted with pBFR combined with exercise using different elastic wraps in terms of material composition and architecture, as well as applying this type of training to healthy (i.e., athletes) and clinical populations (i.e., obese, injured).

## Introduction

Blood flow restriction (BFR) training is characterized by using a relatively light and flexible wrap (i.e., pneumatic cuff, elastic wraps) placed in the proximal region of the upper or lower limbs in order to apply adequate pressure to the limbs capable of restricting blood flow in the muscle due to occlusion of venous blood flow and restricting the arterial blood flow. This type of training has shown an increase in muscle mass, strength and performance in different populations when combined with strength or aerobic training (e.g., older adults, athletes, injured patients) [1–4].

Most studies with BFR training have been conducted using devices capable of regulating the restriction pressure, such as pneumatic cuffs. The researchers in the first BFR training studies arbitrarily used restriction pressures with fixed or progressive values for all subjects [4, 5]. Then afterward in considering individualized pressure prescription, researchers began to use a hand-held Doppler probe together with a pneumatic cuff to find the arterial occlusion pressure (AOP) at rest (“maximum pressure” = 100%) and then prescribed the BFR for physical training based on this value (i.e., 50% of the AOP) [6]. Even though knowing the amount of pressure in mmHg applied to the limbs is very important, especially for clinical and research environments, this may not be a viable option for the general population who exercise in gyms, squares and sports centers. Thinking about this logic, Loenneke and Pujol [7] created the practical blood flow restriction (pBFR) training in 2009, suggesting the use of a 7.6-cm-wide elastic knee wrap (Harbinger Red-Line, Fairfield, CA, USA) as an alternative to the traditional BFR, as it is low cost, affordable and practical.

In this sense, several studies have investigated the acute and chronic effects of pBFR training, but unlike traditional BFR training which seems to present a consensus regarding the prescription of BFR pressure based on AOP, pBFR training studies have used different techniques to apply the pressure/tension exerted by the elastic wrap. Therefore, this Current Opinion article aims to critically and chronologically examine the techniques used to prescribe the pressure exerted by the elastic wrap during pBFR training in an attempt to suggest a valid and safe standard technique for pBFR training in a practical and scientific context.

### Prescription of pBFR Training: Focusing on Methodological Aspects

After Loenneke and Pujol [7] suggested the application of pBFR training, several studies started using pBFR combined with resistance exercise [8, 9] and aerobic exercise [10, 11] in different populations, for example with female and male adults [8–12], and for injured athlete in need of osteochondral fracture rehabilitation [13]. The first studies conducted with resistance exercise combined with pBFR with continuous pressure, showed conflicting data. In the study by Loenneke et al. [8] perceived exertion responses were significantly higher after the first and second set of resistance exercise with pBFR when compared to the same exercise protocol without BFR (control). In contrast, in the study by Loenneke et al. [14] perceived exertion responses were similar between pBFR and control. In both studies, the subjects performed bilateral leg extensions with a load at 30% of 1RM until exhaustion. These contradictory findings seem to demonstrate that the pressure applied during pBFR training may not have been sufficient to adequately restrict arterial blood flow, probably due to the way the elastic bands were wrapped around the limbs (see illustration in Loenneke and Pujol [7]). Furthermore, it was observed that no robust criteria were used for prescribing the elastic wrap (7.6 cm wide), as it was only reported that the elastic wrap was placed by the same investigator to maximize intra-rater reliability.

Additionally, in a study by Yamanaka et al. in 2012 [15], pBFR training with resistance exercise induced an increase in strength and limb girth in athletes. The elastic wrap (5 cm wide) was pulled to overlap 5.08 cm in relation to the initial length of the elastic applied without tension; thus, an arbitrary fixed prescription was used for all individuals. This same technique was later used in several studies [16–18]. It is important to highlight that the aforementioned procedures of the studies [8–15] regarding the prescription of pBFR pressure were performed without knowing what effect was being caused on the arterial and venous blood flow.

Considering this, in 2013, Wilson et al. [19] sought to validate pBFR using the same elastic wraps from previous studies (7.6 cm wide). The authors observed that the elastic consistently resulted in complete vein occlusion when it was tightened on the thigh based on the perceptual response of 7 (moderate pressure without pain) on the tightness scale with 11 descriptors (0–10), but not in the arteries. This way of applying elastic wraps according to the response of a 7 out of 10 on the perceived tightness scale has subsequently been used in several studies [20–28]. However, applying the elastic wrap just for the perception of tightness, seems to be a limited prescription for pBFR, since there is no guarantee that the researcher or trainer will equally restrict the elastic segment in all training sessions.

Later studies tried to elucidate this concern. Bell et al. [29] analyzed subjects' levels of perceived tightness during gradual inflation of a pneumatic cuff in the upper and lower limbs. The pressures found were equivalent to 92% and 73% of the AOP for the upper and lower limbs, respectively, when the subjects answered 7 on the perceived tightness scale, and when they answered 10 on the scale it was 126% and 106% of the AOP for the upper and lower limbs, respectively. It is worth noting that restrictive pressures above 80% of the AOP can be considered high and in most cases are not recommended for BFR training [30]. Using a similar protocol, Bell et al. [31] analyzed the reliability of applied pressure when asking participants to rate a 7 out of 10, over 3 separate visits. The findings reported that the perceived tightness scale does not provide reliable estimates of relative pressures over multiple visits. Additionally, Bell et al. [32] observed that 5 min and 24 h after a conditioning protocol with specific pressures, subjects were unable to accurately estimate the applied pressures. Importantly, all three studies [29, 31, 32] used pneumatic cuffs to measure the reproducibility or validity of perceived tightness; however, pBFR training uses non-inflatable elastic wraps.

In this sense, our laboratory developed a method in 2016 [33] to prescribe the pressure exerted by the elastic based on the circumference of the upper and lower limbs, which, according to previous studies, seems to be the greatest predictor for determining the AOP [34–36]. According to procedures described and illustrated by Aniceto [33] and Aniceto et al. [37], an elastic knee wrap 7.6 cm wide and 94 cm long (Harbinger Red-Line, Fairfield, CA, USA) was adapted by placing 5 cm of Velcro on the ends at the front and back, thereby enabling better fixation on the limbs. In turn, with the purpose of finding a circumference percentage which reflected a perceived tightness of 7 (moderate pressure without pain) on the scale proposed by Wilson et al. [19], circumference measurements of the upper and lower limbs were performed at rest at different times, and then the elastic wrap was applied using the same circumference of the segment (arm or thigh) for 30 s in order to familiarize the subject with the perception equivalent to a rating of 0 (no pressure; low anchorage); then, after 1 min the elastic was stretched to the maximum on the limb for 30 s so that the subject could experience a rating of 10 (intense pressure with pain; high anchorage).

Taking as a reference the circumference of the arm and thigh (100%), the subjects were then randomly assigned to four pBFR conditions (15%, 20%, 25% and 30%) and answered a number on the scale which represented the perceived tightness. For example, a subject with an arm circumference of 30 cm in the 20% pBFR condition had the elastic marked with adhesive tape at 24 cm and this 6 cm restriction was applied to the arm; thus, the elastic was stretched up to 24 cm in the arm with a circumference of 30 cm. Our data showed that most subjects responded 6 to 7 on the scale when they had the elastic restriction at 25% of the circumference for the upper limb and 30% for the lower limb. The reliability coefficients (ICC) for these tightness perception measures were 0.74 (*P* = 0.014) for the arm and 0.86 (*P* = 0.001) for the thigh.

Unlike the previously reported techniques, in 2017, Behringer et al. [38] introduced a technique based on the elasticity of the elastic wrap. They pulled the wraps (13-cm wide) maximally (100% stretch) around the participants’ thighs and marked them at each quarter of every winding. Then, the wraps were removed and reapplied with 75% of their maximum stretch. The authors justified using this technique because they observed that the length remained fairly constant after initially stretching the elastic wraps, so they decided to stretch the knee wraps before their first use to reduce the effect of material slackening at later time points of the study. The authors additionally used an ultrasound system to ensure that the arterial blood flow was not occluded at this pressure. This technique was later used in a few studies [39, 40]. Despite the precautions used by the authors, this technique has some limitations. The composition and mechanical properties of the elastic affect how much the elastic can be stretched, so in some cases with stiffer elastics this technique may be inappropriate. In addition, several researchers may apply different force when stretching the elastic, and thus achieve different lengths of elastic stretching. These issues make it difficult to apply the technique and compare studies.

In 2018, Abe et al. [41] used a similar procedure to our laboratory in relation to the pBFR pressure prescription based on the limb circumference, and demonstrated that the brachial arterial blood flow was not different between the elastic wrap (5-cm wide; custom built (no manufacturer)) and nylon pneumatic cuff (5-cm width; 60 cm length; SC5 Hokanson, Belleview, WA, USA), respectively, when subjects were assessed for low pressure BFR (10% of the arm circumference vs. 40% of AOP) and high pressures (20% of the arm circumference vs. 80% of the AOP). The results indicate that an elastic wrap pulled to 10% and 20% of its arm circumference decreases brachial artery blood flow in a pressure-dependent manner. These data reinforce that prescribing the pressure exerted by the elastic for pBFR training based on the circumference of the limbs seems to be a valid and effective prescription.

Another major issue related to pBFR prescription, and which influences the pressure percentage is related to the elastic’s material composition and architecture. We use 25% and 30% elastic restraint percentages in our laboratory [33, 37, 42], while other laboratories use 10% and 20% [41], and 15% [18], based on the circumference of the limb at rest. This difference is related to the elastics used; the elastic knee wrap (Harbinger Red-Line, Fairfield, CA, USA; 7.6 cm width) used in our laboratory [33, 37, 42] and in various studies [8–10, 13, 19] consists of a single layer of elastic rubber, making it possible to stretch the elastic to 35% of the initial length in a practical way. On the other hand, the elastic wrap (Custom Built (No Manufacturer); 5 cm width) used in the study by Abe et al. [41] consists of three layers of elastic rubber with a practical possibility of stretching up to approximately 30% of the initial length.

The material organization and general structure of the elastic wraps determine its ability to resist deformation. In this sense, according to Hamill et al. [43] a stress–strain analysis can be performed to verify how a material changes over time, how it reacts to different force applications and the absence of daily stress application. In this perspective, according to a thesis published by Gomes [44], the resistance of two elastic knee wraps (hard vs. soft—Maba Murphy Confecções Ltda, Brazil) was tested with the same composition (70% polyester and 30% elastodiene) and the same dimensions (2 m long and 8 cm wide); however, through digital photographs he observed that the hard elastic knee wrap presents twofold smaller spacing between wefts than the soft elastic knee wrap, and therefore the hard elastic presented 42.3% more elasticity compared to the soft due to these structural characteristics. On the other hand, the soft elastic wrap endured 41.15% more deformation than the hard elastic wrap when reaching the maximum elasticity point before rupture. The author concluded that the spacing between wefts was decisive for the elastic bands to present differences in the flow point (end of the elastic zone and beginning of the plastic zone) and in the breaking point, and the polyester was responsible for the maximum tension limit of the analyzed elastic knee wraps.

In this stress–strain analysis perspective, Abe et al. [41] simply and practically reported a calibration procedure for the elastic. The authors vertically fixed one end of the elastic on the wall and placed a load (tension) at the other end to observe the elastic deformation assuming that there is a linear relationship between stress and deformation in this type of material. Thus, it was observed that the elastic stretched 2.7% of the initial length for each 1 kg of load, maintaining this linear ratio until it stretched to around 25%. Accordingly, it is suggested that this type of procedure is performed before training sessions to observe the wear of the elastic wrap. In addition, it is recommended that the elastic length is measured before and after the load is removed in order to verify the mechanical elasticity property of the elastic wrap. With these measures it is possible to check the extensibility and elasticity properties of the elastic wrap and thus make the decision to change it or create a correction factor.

The researchers in these studies carry out reproducibility measures, report the calibration procedure results, as well as (if possible) present the composition and mechanical properties/characteristics of the elastic wrap structure (i.e., elasticity coefficient) [45] in order to enable better comparison between studies and reproduce results. Additionally, in a training program that uses multiple exercise sessions, it is suggested after the measurement of the limb circumference, that a transverse mark is made on the limb with a permanent marker to delimit the height of the elastic wrap (upper edge), in this way, maximize the reproducibility of elastic application. After applying the elastic wrap on the proximal portion of the limb, it is essential to check that the arterial blood flow is not occluded. The portable vascular Doppler can be used with the probe placed in the brachial or tibial artery. This verification must be carried out in the three positions: supine, sitting and standing, given the differences between the positions in relation to AOP [46].

### Safety

Few studies in the literature have been concerned with analyzing variables which could verify the safety of pBFR training [30, 47]. In analyzing muscle damage, Wilson et al. [19] demonstrated that soreness, power and muscle swelling were similar between low-load resistance exercise with and without pBFR; in addition, Behringer et al. [38] demonstrated that after 6 weeks of sprint training, the heart-type fatty acid-binding protein (h-FABP) was significantly lower in the group that trained with pBFR than in the control group, with similar responses between groups regarding cortisol. Additionally, studies have found similar acute pain responses between low-load resistance exercise with and without pBFR [26, 40]; furthermore, the high-load resistance exercise induces greater pain scores than low-load resistance exercise with pBFR [26]. Considering cardiovascular events, studies have compared high-load resistance exercise with low-load resistance exercise with pBFR, noting that post-exercise acute responses are similar between the exercise protocols, in relation to autonomic modulation [25], as well as on arterial stiffness and brachial systolic or diastolic blood pressure [24].

These findings lead us to think that pBFR training in healthy individuals is safe, and it seems that side effects or adverse events are minimal, with risks being minimized when the practitioner or researcher is well trained using appropriate methods in applying the elastic wrap or cuff. According to Brandner et al. [47], most often the side effects caused by traditional BFR training seem to be associated with high pressure applied by the cuff (~ 200 mmHg) or when thin cuffs (~ 3 cm) are used. Previous studies have reported that wider cuffs require a lower pressure to occlude blood flow compared with narrower cuffs [34, 36, 48, 49]. Additionally, higher systolic and diastolic blood pressures have been reported when using narrower cuffs in comparison with wider cuffs [50].

Based on this information, we believe the concerns are the same with pBFR training, meaning that high pressures and thin elastic bands should be avoided. However, some possible contraindications of pBFR training should be taken into consideration, such as venous thromboembolism, peripheral vascular disease, unstable hypertension and pregnancy [51]. In this sense, the scoring system proposed by Nakajima et al. [51] or the clinical screening tool proposed by Kacin et al. [52] assist in tracking risk factors; thus, both tools can be used to assess whether the individual has any contraindication to perform pBFR training. In addition, it is recommended to check the ankle brachial index (0.90 ≤ ABI ≤ 1.40) as it is a predictor of cardiovascular disease, and most studies with pBFR do not perform this measurement.

## Conclusion and Perspectives

The literature demonstrates that in most cases the elastic wrap can replace the pneumatic cuff traditionally used for BFR training. Moreover, it is possible to apply different pressures with the elastic wrap to the individual, making it possible to perform pressure progression in the pBFR training over time. In summary, five techniques were found chronologically to apply the elastic wrap during pBFR training: (i) application-based technique by the same investigator to maximize intra-rater reliability; (ii) absolute overlap of the elastic tightened to a fixed value (for example, 5.08 cm, 7.60 cm) of the initial application without tension; (iii) according to the perceived tightness scale; (iv) relative overlap of the elastic, in which it was stretched to the maximum (100%) in the segment, removed and then reapplied with 75% of the maximum stretch; and (v) relative overlap of the elastic based on the circumference of the limbs. Advantages and disadvantages can be observed given the evolution of the techniques used in the studies since 2009, but several studies have shown that limb circumference seems to be the greatest predictor of AOP. Therefore, we reinforce that applying the pressure exerted by the elastic for pBFR training based on the circumference of the limbs is an excellent, valid and safe technique.

In this perspective, it is suggested to use 10–30% elastic restraint percentages based on the limb circumference at rest for pBFR training, depending on the type of elastic, experience and training objective, and also thinking about the individual’s safety and adherence to the training program. Regarding the width of the elastic wrap, we recommend using 5 to 10 cm in width for upper and lower limbs. It is worth reinforcing the need for elastic calibration before exercise sessions, especially in chronic studies. However, future studies should be conducted with pBFR combined with exercise using different elastic wraps in terms of material composition and architecture, as well as applying this type of training to healthy (i.e., athletes) and clinical populations (i.e., obese, injured).

## Data Availability

Not applicable.

## Abbreviations

BFR: Blood flow restriction
AOP: Arterial occlusion pressure
pBFR: Practical blood flow restriction
h-FABP: Heart-type fatty acid-binding protein
ABI: Ankle brachial index
